# Role of Cytokine-Induced Glycosylation Changes in Regulating Cell Interactions and Cell Signaling in Inflammatory Diseases and Cancer

**DOI:** 10.3390/cells5040043

**Published:** 2016-11-29

**Authors:** Justine H. Dewald, Florent Colomb, Marie Bobowski-Gerard, Sophie Groux-Degroote, Philippe Delannoy

**Affiliations:** 1 Structural and Functional Glycobiology Unit, UMR CNRS 8576, University of Lille, Villeneuve d’Ascq 59655, France; justine.dewald@etudiant.univ-lille1.fr (J.H.D.); sophie.groux-degroote@univ-lille1.fr (S.G.-D.); 2 Department of Cellular and Molecular Physiology, Institute of Translational Medicine, University of Liverpool, Liverpool L69 3GE, UK; florent.colomb@liverpool.ac.uk; 3 Institut Européen de Génomique du Diabète (EGID), Centre hospitalier universitaire (CHU) de Lille, Institut Pasteur de Lille, Institut national de la santé et de la recherche médicale (INSERM) U1011, University of Lille, Lille 59000, France; marie.bobowski@hotmail.fr

**Keywords:** cytokines, glycosylation, glycosyltransferases, *O*-glycans, mucins, gangliosides, RTKs, cell signaling, sialyl-Lewis^x^

## Abstract

Glycosylation is one of the most important modifications of proteins and lipids, and cell surface glycoconjugates are thought to play important roles in a variety of biological functions including cell-cell and cell-substrate interactions, bacterial adhesion, cell immunogenicity and cell signaling. Alterations of glycosylation are observed in number of diseases such as cancer and chronic inflammation. In that context, pro-inflammatory cytokines have been shown to modulate cell surface glycosylation by regulating the expression of glycosyltransferases involved in the biosynthesis of carbohydrate chains. These changes in cell surface glycosylation are also known to regulate cell signaling and could contribute to disease pathogenesis. This review summarizes our current knowledge of the glycosylation changes induced by pro-inflammatory cytokines, with a particular focus on cancer and cystic fibrosis, and their consequences on cell interactions and signaling.

## 1. Introduction

Glycosylation is one of the most important modifications of proteins and lipids, and cell surface glycoconjugates are thought to play important roles in a variety of biological functions including cell-cell and cell-substrate interactions, bacterial adhesion, cell immunogenicity and cell signaling. Glycan structures are depending on the cell type, developmental stage and cell differentiation, and are modified in pathologic states including cancers and inflammatory diseases such as cystic fibrosis or bowel diseases. The modifications of cell glycosylation observed in cancers mainly affect the outer part of glycans, leading to the expression of cell surface antigenic sialylated structures that are strongly associated with a poor prognosis in certain tumors [1,2,3]. For example, sialyl-Lewis^a^ (sLe^a^) and sialyl-Lewis^x^ (sLe^x^) antigens are abnormally found on glycoproteins and glycolipids in several types of solid tumors and sLe^x^/selectin interactions are clearly involved in metastatic progression of gastric [4], lung [5] and prostate [6] cancers. These changes of cell glycosylation are mainly supported by the deregulation of the expression of glycosyltransferases (GTs) genes implicated in terminal glycosylation. Notably, several sialyltransferases were demonstrated to be involved in cancer progression (for review: [7]).

Inflammation is also known to modify the glycosylation pattern of glycolipids and glycoproteins. It is well known that the glycosylation of acute-phase proteins is subjected to marked changes during acute and chronic inflammation [8]. Moreover, several GT genes were shown to be regulated at the transcriptional level by pro-inflammatory cytokines such as tumor necrosis factor (TNF) or interleukin 6 (IL-6) [9,10], leading to the expression of sialylated structures such as sLe^x^ also observed in cancers. In parallel, changes in cell surface glycosylation are known to regulate cell signaling by different mechanisms. In particular, within glycolipid-enriched microdomains, gangliosides act as fine regulators of Receptor Tyrosine Kinase (RTK) signaling [11] and changes in ganglioside composition of cell membrane are implicated in several diseases including cancers from neuro-ectoderm origin [12]. Cancer tumor development is also associated with an important inflammation and the presence of pro-inflammatory cytokines at the tumor site that can reinforce the progression of the disease. Finally, cytokines can also be involved in altered glycosylation observed in several pathologies with sustained inflammation. In particular, in lungs from patients suffering from cystic fibrosis (CF), increased levels of pro-inflammatory cytokines are responsible for *O*-glycosylation alterations exhibited by CF bronchial mucins.

The aim of this review is to summarize our current knowledge of the relationship between inflammation, pro-inflammatory cytokines, glycosylation and progression of inflammatory diseases and cancers.

## 2. Regulation of Mucin *O*-Glycosylation by Pro-Inflammatory Cytokines

### 2.1. Glycosylation and Sulfation Alterations of Mucin Type O-Glycans in Cystic Fibrosis

#### 2.1.1. Physiopathology of Cystic Fibrosis

Cystic fibrosis (CF) is the most frequent autosomal recessive disease among Caucasians. It is characterized by a general exocrinopathy, but the major problem for CF patients is bronchial mucus hypersecretion and severe chronic lung infection, mostly by *Pseudomonas aeruginosa* (*P. aeruginosa*). CF is due to mutations in the cystic fibrosis transmembrane conductance regulator (*CFTR*) gene, encoding an apical membrane chloride channel [13] that also affects several other epithelial channels or transporters [14]. More than 2000 different mutations of *CFTR* have been described [15], which can affect CFTR protein synthesis or function at different stages. In CF patients, CFTR, which is normally expressed at the apical membrane of epithelial cells (bronchial, pancreatic, intestinal…), is therefore absent or defective. The major mutation, found in 90% of CF alleles, is ΔF508. In that case, the deletion of a Phe residue in position 508 induces the production of an abnormally folded CFTR protein, which is subsequently degraded in the endoplasmic reticulum, preventing therefore CFTR protein to be targeted at the apical cell surface. The defective chloride transport leads to abnormal ion and water transport across the epithelia, which induce dehydration of secretions (mucus) and obstruction of exocrine glands. The resulting clinical outcomes are chronic airway obstruction and infection, pancreatic insufficiency, intestinal malabsorption and sterility. Since the lung disease is the major cause of premature death, abnormalities in CF bronchial mucus and their major component (the bronchial mucins) have been widely studied. Mucin-type *O*-glycans are characterized by the linkage of an *N*-acetylgalactosamine (GalNAc) residue to selected serine or threonine residues in glycoproteins, and elongated by different monosaccharides including galactose (Gal), *N*-acetylglucosamine (GlcNAc), fucose (Fuc) and sialic acids by the action of various glycosyltransferases. Mucins are responsible for the rheological properties of the mucus layer and the modifications of these heavily glycosylated *O*-glycosylproteins could modify mucus properties including mucociliary clearance and lead to specific bacterial colonization and infection.

#### 2.1.2. Biosynthesis of Human Bronchial Mucins and Their Alterations in CF

##### 2.1.2.1. Bronchial Mucin Biosynthesis

**Figure 1:**
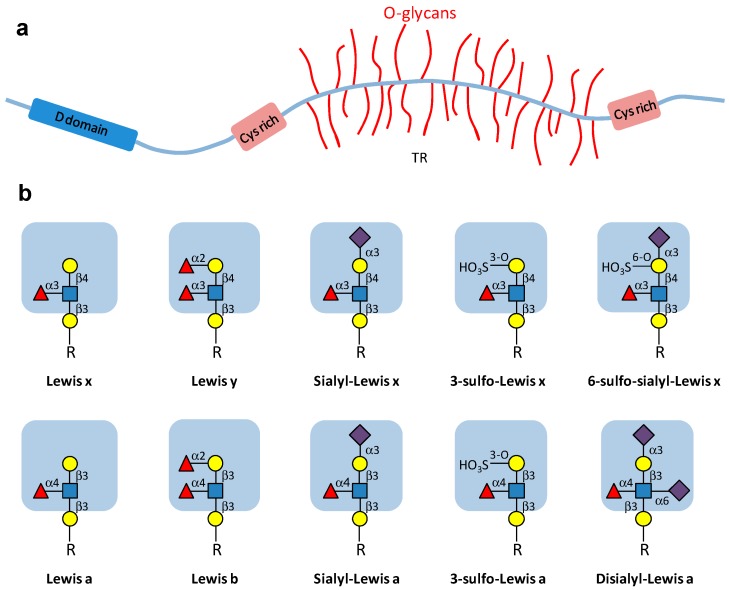
(**a**) Schematic drawing of a secretory mucin type glycoprotein constituted by a MUC protein backbone (apomucin) carrying numerous *O*-glycans attached to Thr or Ser residues contained in the variable number of tandem repeat (TR) domains. Cysteine-rich (Cys rich) domains at the ends of the apomucin are involved in the formation disulfide bonds to form larger polymeric structures. D domains share similarities to von Willebrand factor and are also involved in polymerization. Adapted from Brockhausen et al. [21]. (**b**) Major types of carbohydrate structures present at the periphery of bronchial mucin *O*-glycan chains. Sialylated and/or sulfated derivatives of the Lewis epitope can be present on terminal type 1 (Galβ1-3GlcNAc) or type 2 (Galβ1-4GlcNAc) disaccharides. Symbols used for the different monosaccharides are the following: 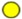: Gal; 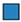: GlcNAc; 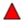: Fuc; 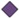: Neu5Ac; HO_3_S: sulfate. R: *O*-glycan chain.

Bronchial mucins, the major component of the bronchial mucus, are a family of heavily *O*-glycosylated high molecular weight glycoproteins which fulfill many biological functions: they serve as a protective barrier for the lung epithelium while they also modulate cell signaling and survival [16]. Mucins can be divided into two major families: secreted gel-forming mucins, which are the major components of the bronchial mucus (Figure 1a), and membrane-bound mucins. The mucin genes (*MUC*), mostly expressed in the respiratory tract, encode membrane-bound mucins (MUC1, MUC4) as well as secreted mucins (MUC5AC, MUC5B, MUC19, MUC2, MUC7). MUC5AC and MUC5B are the major components of the bronchial mucus, and their synthesis and secretion is modulated by inflammatory factors such as neutrophil elastase, bacteria, and cytokines [17]. Modifications in bronchial mucin biosynthesis, secretion and *O*-glycosylation have been demonstrated in several pathologies, including lung cancer, but also chronic bronchitis, asthma and CF as we will discuss in Section 2.1.2.2 [18,19,20]. In most cases, these changes induce unfavorable modifications of mucus viscosity, impairing of mucociliary transport, and increasing infection by viruses and bacteria, which use modified sugar chains as receptors.

Mucin carbohydrate chains are synthesized by the sequential action of Golgi-localized glycosyltransferases. The first step of mucin *O*-glycosylation is accomplished by one of the 20 uridine-diphosphate-*N*-acetylgalactosamine (UDP-GalNAc): polypeptide-α-*N*-acetylgalactosami­nyltransferases (ppGalNAcT), which catalyzes the transfer of an initial GalNAc residue to the hydroxyl group of a serine or threonine from the apomucin [22]. This linkage provides the starting point for branching oligosaccharide chains. The elongation of the chains leads to various linear or branched extensions. Mucin type *O*-glycans contain GalNAc, Gal, GlcNAc, Fuc, sialic acids (*N*-acetylneuraminic acid, Neu5Ac) residues and can also be sulfated. Their non-reducing end may bear different terminal structures, such as histo-blood groups H, A or B determinants, Lewis^a^, Lewis^b^, Lewis^x^ or Lewis^y^, as well as sialyl- and/or sulfo-Lewis^a^ or Lewis^x^ determinants [23] (Figure 1b).

##### 2.1.2.2. Alterations of Mucin Glycosylation and Sulfation of CF: Influence on *P. aeruginosa* Adhesion

Glycosylation defects of glycoconjugates from CF cells or secreted by CF patients are widely described. Bronchial mucins purified from the sputum of CF patients are more sulfated, sialylated and fucosylated than those from non-CF individuals. Numerous studies have shown an increased sulfation of salivary and intestinal mucins from CF patients [24,25,26]. The structural determination of numerous neutral and acidic *O*-glycan chains from bronchial mucins purified from CF or chronic bronchitis patients has allowed identification of the precise carbohydrate structures over-expressed in CF [23,27,28]. CF bronchial mucins contained more sialylated and sulfated *O*-glycans compared with non CF mucins, with increased amounts of sLe^x^ and 6-sulfo-sialyl-Lewis^x^ (6-sulfo-sLe^x^) structures. Interestingly, the sLe^x^ structure is over-expressed on bronchial mucins from severely infected CF patients, but also on bronchial mucins from infected patients with other lung pathologies, such as chronic bronchitis [29]. These major differences in *O*-glycan composition (and possibly density) in CF compared with non CF individuals could deeply modify interactions of *P. aeruginosa* with bronchial mucins. Indeed, sLe^x^ and 6-sulfo-sLe^x^ determinants have been described as preferential ligands for *P. aeruginosa*, and their over-expression could therefore contribute to the specificity and the chronicity of CF airways infection by these bacteria [30,31].

Interestingly, it seems that glycosylation of membrane-bound CF glycoconjugates and secreted glycoconjugates are differently affected. In particular, membrane glycoconjugates from CF epithelial cells are less sialylated than wild type cells [32]. Since mucins secreted by CF patients are oversialylated, factors others than the *CFTR* deficiency are likely responsible for the altered glycosylation (sialylation) of CF mucins. Since airway mucin-secreting cells express no or very low CFTR amounts, these glycosylation modifications cannot be directly linked to defective *CFTR* expression. Because CF is characterized by chronic and unresolved lung inflammation, and since there is an abundant literature on the effects of inflammation on glycosylation [8], the link between lung inflammation in CF patients and glycosylation/sulfation of bronchial mucins has been studied.

### 2.2. Inflammation in CF and Altered Mucin Glycosylation

CF lung disease is characterized by vigorous and unresolved inflammation, with elevated pro-inflammatory and decreased anti-inflammatory cytokines, and increased numbers of immune cells. This hyper-inflammation is now recognized as a leading cause of lung tissue destruction in CF.

#### 2.2.1. The Vicious Circle of Inflammation/Infection in CF Airways

**Figure 2:**
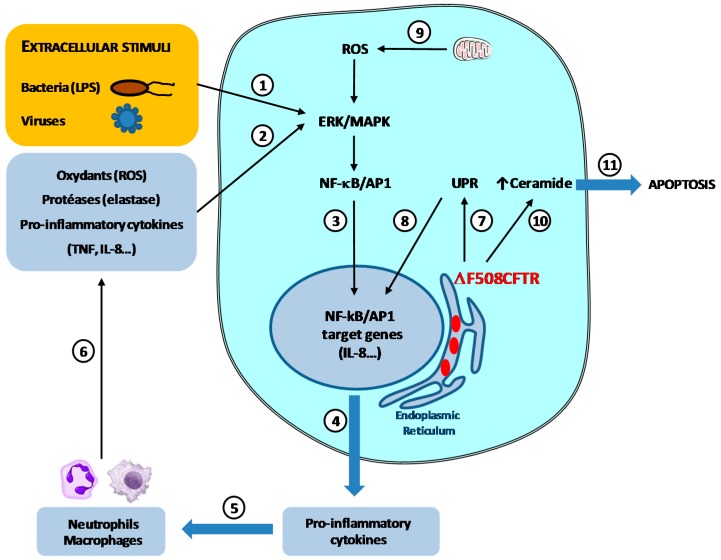
Extracellular and intracellular triggers of inflammation in CF airways cells. Pathogens, such as bacteria and viruses ①, as well as increased levels of pro-inflammatory cytokines, protease and ROS chronically present in CF airways ② lead to a constant stimulation of the ERK/MAPK pathway, resulting in NF-κB/IκB complex dissociation and NF-κB activation ③. NF-κB activation results in increased synthesis of pro-inflammatory cytokines, such as TNF, IL-8, IL-17 ④, leading to increased neutrophil influx ⑤, which contributes to the vicious inflammatory circle by releasing high amounts of oxidants, proteases and pro-inflammatory cytokines ⑥. Retention of ΔF508CFTR in the endoplasmic reticulum of airway epithelial cells induces unfolded protein response (UPR) ⑦, cell stress, NF-κB constitutive activation ⑧ and increased pro-inflammatory cytokines production ④, perpetuating the inflammatory response in the airways. In CF cells, the increased reactive oxygen species (ROS) production by mitochondria ⑨, as well as increased intracellular ceramide levels ⑩ contribute to NF-κB activation ③ and sustained inflammation, as well as cell apoptosis ⑪.

In most CF patients, early death is linked to a progressive loss of functional lung tissue due to a combination of airway obstruction, infection, and inflammation. Results suggest that CF airway inflammation occurs very early in life, and could even precede infection: increased amounts of neutrophils, neutrophil elastase, and pro-inflammatory cytokines concentrations (especially IL-8) can be detected in broncho-alveolar lavages (BAL) of young CF children (under 6 months) in the absence of common CF-related pathogens [33]. The cause of this early inflammation and how this is related to CFTR deficiency or CF related bacteria such as *P. aeruginosa* is not clearly understood. BAL and sputum from adult CF patients also contain increased amounts of pro-inflammatory cytokines such as TNF, IL-1, IL-6, IL-8, and IL-17, compared to non-CF controls [34,35]. In addition, it has been shown that different types of CF cells secrete increased amounts of pro-inflammatory cytokines (IL-1β, IL-6, IL-8), whereas anti-inflammatory cytokine IL-10 is decreased [36]. Moreover, blood and lung neutrophils from CF patients synthesize high levels of IL-8, which are even increased by lipopolysaccharide (LPS) treatment, suggesting that infection can contribute to perpetuating the “vicious circle of inflammation” in CF [37,38] (Figure 2). In this connection, elevated levels of pro-inflammatory cytokines IL-17A and IL-17F were found in the sputum of CF patients, related to *P. aeruginosa* colonization [35]. Since human bronchial epithelial cells treated with IL-17A and IL-17F show increased secretion of IL-8 via the mitogen-activated protein kinase (MAPK) pathways, IL-17A and IL-17F are likely involved in pro-inflammatory gene expression and the pro-inflammatory cytokine network involved with CF pathogenesis. However, in spite of increased neutrophil influx, CF airways fail to eradicate bacteria efficiently.

The link between a defective CFTR protein and the exaggerated/deregulated inflammatory response in CF patients/cells is still not fully understood. Weber et al. have demonstrated that ΔF508CFTR mutation, with impaired folding and activity, accumulates in the endoplasmic reticulum, resulting in nuclear factor-κB (NF-κB) activation and increased IL-8 synthesis, even in the absence of bacteria [39]. Other studies suggest that non functional CFTR affects the lung antioxidant defenses in mice and may contribute to the exaggerated inflammatory response observed in CF [40]. Defective CFTR leads to an increase in the level of reactive oxygen species (ROS) and mitochondrial oxidative stress in the lungs of CF mice. ROS could therefore also be involved in the initiation or the chronicity of inflammation in CF cells. Increased ROS levels in CF cells could activate the MAPK pathway, which can induce the expression of inflammation-related genes [41]. Another key signaling molecule, ceramide, is increased in the lower airway epithelium of people with advanced cystic fibrosis lung disease [42]. In CFTR deficient mice, the age-dependent accumulation of ceramide leads to pulmonary inflammation, respiratory epithelial cells death, and susceptibility to severe *P. aeruginosa* infections [43]. Ceramide acts as a second messenger in activating the apoptotic cascade, the NF-κB pathway as well as pro-inflammatory cytokines up-regulation, contributing to chronic inflammation and increased neutrophils and macrophages in the lungs (Figure 2).

In conclusion, a number of studies suggest that CF airway epithelial cells display abnormalities in signaling and intracellular processes which increase the synthesis of inflammatory mediators. Several transcription factors including NF-κB are activated and the accumulation of misfolded CFTR in the endoplasmic reticulum triggers cell stress and apoptosis, leading to exaggerated and ineffective airway inflammation. NF-κB activation induces an imbalance between pro and anti-inflammatory cytokines (TNF, IL-1, IL-6, IL-8, IL-17 vs. IL-10). Increased pro-inflammatory cytokines expression is associated with additional activation of NF-κB transcription factor and AP-1, controlled by IκB kinase/ERK pathway (Figure 2). ROS and ceramide both also activate NF-κB and AP-1, contributing to the vicious circle of inflammation [44], which is perpetuated by intense lung neutrophilic inflammation and protease release (reviewed in [45]). The sustained inflammation in CF lungs could be responsible for some of the modifications of glycosylation and sulfation exhibited by CF bronchial mucins, leading to an increased and specific *P. aeruginosa* infection.

#### 2.2.2. Influence of Pro-Inflammatory Cytokines on the Expression and Activity of GTs and SulfoTs

The pro-inflammatory cytokine TNF, present in high amounts in BAL of CF patients, can increase the expression and activities of some sialyl-, fucosyl- and sulfotransferases in human bronchial explants as well as in the human respiratory glandular cell line MM-39 [9,46]. In human bronchial mucosa, TNF increases α2,3-sialyltransferase activity and the expression of α2,3-sialyltransferase *ST3GAL3* and *ST3GAL4* genes, as well as GlcNAc-6-*O*-sulfotransferase and Gal-3-*O*-sulfotransferase activities. These results could explain the oversialylation and sLe^x^ over-expression on human airway mucins secreted by patients with severe lung infection such as those with CF [9]. IL-6 and IL-8 are also present in high amount in BAL fluids of CF patients. We have shown in a bronchial explants model that IL-6 or IL-8 treatment results in an increased expression of α1,3/4-fucosyltransferases genes (*FUT3*, *FUT11*), α2,3-sialyltransferases genes (*ST3GAL4* and *ST3GAL6*) and GlcNAc-6-*O*-sulfotransferases genes (carbohydrate sulfotransferase *CHST4* and *CHST6* gene). In parallel, high-molecular-mass proteins, including mucin MUC4, carried increased amounts of sLe^x^ and 6-sulfo-sLe^x^ structures [10]. These results indicate that IL-6 and IL-8 may also contribute to the increased sLe^x^ and 6-sulfo-sLe^x^ structures and increased *P. aeruginosa* adhesion on CF airway mucins. Other cytokines present in increased amounts in CF airways, such as IL-17, could also contribute to altered carbohydrate structures exhibited by CF bronchial mucins.

### 2.3. Signaling Pathways Involved in the Regulation of sLe^x^ Biosynthesis by TNF in the Human Bronchial Mucosa; Relation with P. aeruginosa Adhesion

In human bronchial mucosa, TNF-driven inflammation has been shown to be a potent regulator of sLe^x^ over-expression involved in CF pathogenesis. Engagement of TNF-receptor in bronchial cells by TNF cytokine, which is present in increased amounts in inflamed bronchial airways of CF patients, is known to trigger the activation of different signaling pathways, including the NF-κB and MAPK pathways [47]. In the human pulmonary muco-epidermoid carcinoma cell line NCI-H292, the inhibition of the phosphoinositide-phospholipase C (PI-PLC) pathway by U73122 inhibitor is able to repress the TNF-induced over-expression of three genes controlling sLe^x^ biosynthesis on mucin-type *O*-glycans: the α2,3-sialyltransferase gene *ST3GAL4*, the α1,3-fucosyltransferase gene *FUT3* and core 2/4 synthase gene *GCNT4* [48]. These results indicate the involvement of a common signaling pathway regulating these three genes, dependent of the PI-PLC pathway rather than the NF-κB pathway. Further studies concerning the mechanisms involved in *ST3GAL4* gene over-expression by TNF in human bronchial explants and in the A549 lung carcinoma cell line have shown the involvement of Extracellular Signal-Regulated kinases (ERK), p38 and Mitogen- and Stress-activated kinases 1/2 (MSK1/2) in the signaling pathways leading to sLe^x^ and 6-sulfo-sLe^x^ over-expression, while no role of the NF-κB pathway was shown [49].

In parallel, the detailed analysis of the transcriptional expression of *ST3GAL4* has identified the BX transcript as the most expressed *ST3GAL4* transcript in the bronchial mucosa [50]. Moreover, the expression of this specific *ST3GAL4* mRNA is increased by TNF treatment in both the human bronchial mucosa and in A549 lung cancer cell line, and is correlated with sLe^x^ and 6-sulfo-sLe^x^ over-expression on glycoproteins. Interestingly, even if in silico analysis of the *ST4GAL4* genomic sequence have identified putative NF-κB binding site upstream the *ST3GAL4* BX transcription start site (TSS), the only functional TNF responsive element is an intronic sequence downstream the BX TSS [49,50].

Finally, TNF-induced sLe^x^ over-expression is responsible for an enhanced adhesion of *P. aeruginosa* on the NCI-H292 cell line [50]. Indeed, the level of adhesion of two different *P. aeruginosa* strains was increased on NCI-H292 cells treated with TNF. This increased adhesion was shown to be both dependent of the presence of sialic acid and of a functional FliD flagellar cap protein, a sLe^x^-binding protein [31]. Interestingly, a recent study suggests that the *P. aeruginosa* toxin pyocyanin itself is able to modulate sLe^x^ expression on mucins in NCI-H292 cells, leading to an increased binding of *P. aeruginosa* [51]. Since the sialylation and sLe^x^ expression of bronchial mucins secreted by patients suffering from CF or chronic bronchitis is related to the severity of airway infection [29], signaling pathways and molecular mechanisms responsible for TNF-induced over-expression of sLe^x^ in human bronchial mucosa could be of critical importance to understand the links between sustained inflammation and *P. aeruginosa* infection observed in these lung pathologies.

### 2.4. Mucins in Inflammatory Bowel Diseases

Cytokine induced changes in mucin expression and *O*-glycosylation are likely involved in the pathogenesis of inflammatory bowel diseases (IBD). Crohn’s disease and ulcerative colitis (UC), the two forms of chronic human IBD, are both primarily characterized by a chronic inflammation of parts of the gastrointestinal (GI) mucosa. Multiple environmental and genetic factors are involved in the development of these diseases, such as imbalance of the immune system, bacteria of the GI tract, and the intestinal epithelial barrier, whose main component is the mucus layer. The intestinal mucus that forms a protective barrier between the epithelium and the intestinal lumen contains secreted mucins, mostly MUC2. Other types of mucins, such as membrane-bound MUC3, are found at the apical surface of intestinal microvilli but are not considered as being part of the mucus layer. As mucins are present between the intestinal mucosa and the bacterial contents of the bowel, changes in mucin expression, structure and/or glycosylation are likely to influence the protection of the colonic mucosa, bacterial adhesion, and may therefore constitute important factors in the pathogenesis of IBD.

A dysregulated balance between pro- (TNF, IL-1β, IL-8, IL-17) and anti-inflammatory cytokines (IL-4, IL-13), as well as the immuno-regulatory cytokines, has been described in IBD [52]. Importantly, cytokines such as TNF and bacterial components have been shown to influence mucin gene and protein expression in intestinal cell lines [53], as well as in animal models [54,55]. Cytokines such as TNF, IL-6 and IL-8 are known modulators of mucin glycosylation, as described extensively in human bronchial mucosa and in lung cell lines [9,10]. In this connection, several changes have been observed concerning mucins and the mucus layer in UC: the mucus gel layer is thinner than normal [56] and goblet cells responsible for the synthesis of secreted mucins such as MUC2 are reduced in number [57]. Interestingly, alterations in *O*-glycosylation of mucins, especially sialylation and sulfation, have been reported in UC [58,59]. More recently, mass spectroscopy studies have shown that patients with active UC exhibit alterations in MUC2 glycosylation, characterized by an increase in small glycans, especially the Tn (GalNAc-S/T) and sialyl-Tn (NeuAcα2-6GalNAc-S/T) antigens, and lower amounts of larger glycans [60]. There was a significant correlation between glycan expression and both the degree of inflammation and disease course. Interestingly, these antigens are also frequently increased in many cancer types [61]. Increased levels of the TF antigen Galβ1-3GalNAc-S/T were also described in UC, but not on MUC2. Most individuals expressing the TF antigen also exhibited NF-κB activation at epithelial cell surface, indicating a connection between the inflammatory response and the aberrant expression of TF-antigen; and possibly of other glycans [62]. Altogether, data from the literature suggest that glycosylation modifications observed in active UC are rather a consequence of inflammation than linked to a genetic defect. Nevertheless, the mechanisms involved in inflammation-induced mucin glycosylation and sulfation alterations are not described yet, nor are their precise significance on IBD pathogenesis. They could contribute to the decreased protective properties of the colonic mucus barrier observed in IBD. This is in agreement with results obtained from Muc2-deficient mice spontaneously developing colitis and colorectal cancer [63]. Colonic MUC2 carries mostly core 1 (Galβ1-3GalNAc-S/T) and core 3 (GlcNAcβ1-3GalNAc-S/T) based mucin *O*-glycans. The use of animal models allowed to understand to which extent *O*-glycosylation contributes to the development of UC and colorectal cancer. For instance, mice with targeted intestinal deficiency of core 1-derived *O*-glycans developed spontaneous colitis similar to human UC [64]. Moreover, using KO or DKO mice lacking intestinal core 1 and/or core 3 based *O*-glycans, Bergstrom and co-workers showed that high levels of the Tn antigen were found in tissues from DKO mice, which exhibited more severe spontaneous chronic colitis than core 1 KO mice [65]. In addition, both core 1/core 3 DKO and core 1 KO mice developed spontaneous colorectal tumors [65]. Studying mucin properties from these mutant mice showed that core 1 and core 3 derived *O*-glycans are both necessary to maintain the colonic mucus barrier and protect against spontaneous colitis in mice [66]. These data strongly suggest that inflammation-induced glycosylation modifications of intestinal mucins are possible etiological factors in IBD.

## 3. Regulation of Ganglioside Expression by Pro-Inflammatory Cytokines

Gangliosides define a subclass of glycosphingolipids characterized by the presence of at least one sialic acid residues in the carbohydrate moiety. In mammals, they are essential compounds of the outer leaflet of the plasma membrane, where they interact with phospholipids, cholesterol, and transmembrane proteins forming glycolipid-enriched microdomains, also called lipid rafts. Gangliosides were demonstrated to be central molecules in the plasma membrane involved in cell adhesion, proliferation, and recognition processes, as well as in the modulation of signal transduction pathways [67,68]. These different functions are mainly supported by the glycan moiety, and changes in the structure of gangliosides can occur under pathological conditions, including neurodegenerative disorders and cancers [69,70,71]. In particular, the neo-expression of disialogangliosides has been demonstrated in several neuroectoderm-derived tumors in which they play a key role in invasion and metastasis [72], making disialogangliosides attractive target molecules for cancer immunotherapy [73,74].

### 3.1. Biosynthesis of Gangliosides

**Figure 3:**
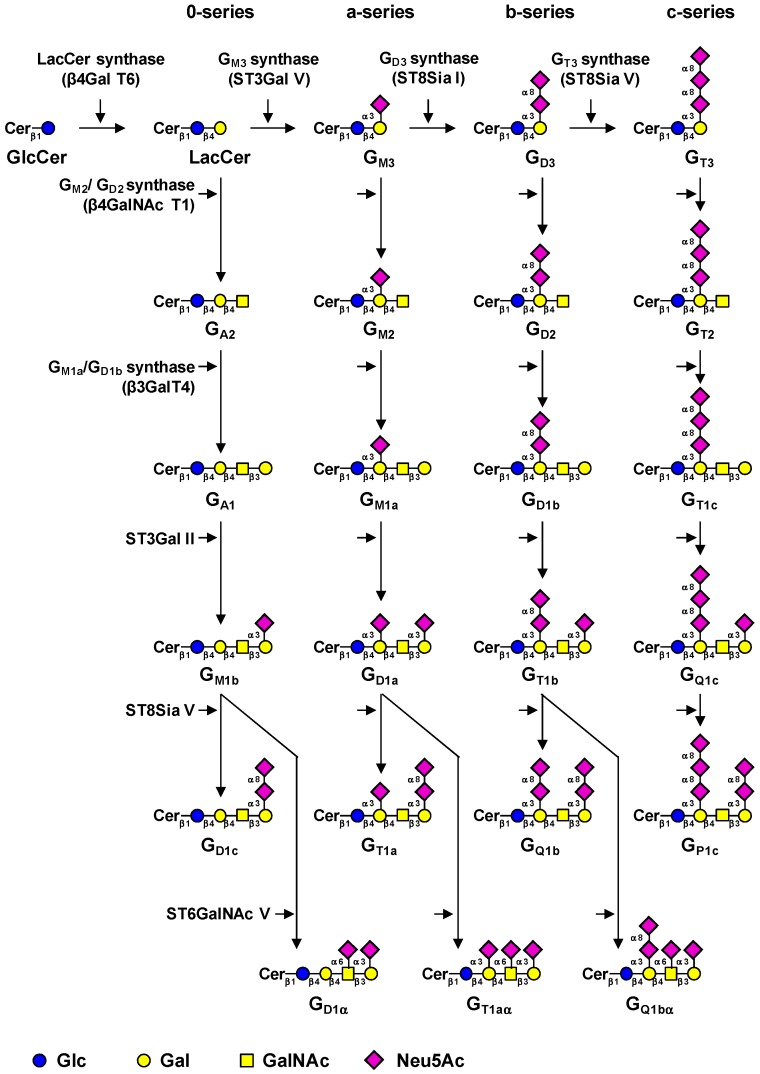
Biosynthesis pathways for gangliosides. Gangliosides are synthesized by stepwise addition of monosaccharides to ceramide (Cer). Ceramide is the acceptor for UDP-Glc: ceramide β-d-glucosyltransferase. Extension of GlcCer occurs by the action of UDP-Gal: GlcCer β1,4-galactosyltransferase to make lactosylceramide (LacCer). The action of ST3Gal V (GM3 synthase), ST8Sia I (GD3 synthase), and ST8Sia V (GT3 synthase) leads to the biosynthesis of the precursors of a-, b-, and c-series gangliosides, respectively. The 0-series gangliosides are directly synthesized from LacCer. Elongation is performed by the sequential action of *N*-acetylgalactosaminyltransferase (β4GalNAc T1), galactosyltransferase (β3Gal T4), and sialyltransferases (ST3Gal II and ST8Sia V). α-Series gangliosides derive from the action of ST6GalNAc V on G_M1b_, G_D1a_, or G_T1b_. The code names of gangliosides are according to Svennerholm [84]. Data from Bobowski et al. [85].

The biosynthesis of gangliosides is a step-by-step process starting in the cis-Golgi by the transfer of a glucose residue onto ceramide (Cer) by the UDP-Glc: *N*-acylsphingosine β-d-glucosyl­transferase (GlcCer synthase) encoded by the *UGCG* gene [75]. The GlcCer synthase is highly specific for ceramide and can be inhibited by d,l-threo-1-phenyl-2-palmitoylamino-3-morpholino-1­propanol (PPMP), blocking the synthesis of almost all glycosphingolipids [76]. The next step consists in the galactosylation of GlcCer by the UDP-Gal: GlcCer β1,4-galactosyltransferase (LacCer synthase) to form the lactosylceramide (LacCer) [77,78]. The transfer of sialic acid residues to LacCer is then catalyzed by the different sialyltransferases: ST3Gal V (GM3 synthase), ST8Sia I (GD3 synthase), and ST8Sia V (GT3 synthase), all showing high specificity toward glycolipid substrates [79]. The human ST3Gal V was shown to use only LacCer as an acceptor substrate to synthesize G_M3_ (II^3^Neu5Ac-Gg_2_Cer) [80]. The GD3 synthase (GD3S) ST8Sia I is also highly specific to G_M3_ [81], but the human enzyme was also shown to use G_D3_ (II^3^Neu5Ac_2_-Gg_2_Cer) to synthesize G_T3_ (II^3^Neu5Ac_3_-Gg_2_Cer) [82]. The human ST8Sia V exhibits broader enzymatic activity toward gangliosides, using G_D3_, but also G_M1b_ (IV^3^Neu5Ac_1_-Gg_4_Cer), G_D1a_ (IV^3^Neu5Ac_1_II^3^Neu5Ac_1_-Gg_4_Cer), or G_T1b_ (IV^3^Neu5Ac_1_II^3^Neu­5Ac_2_Gg_4_Cer) as acceptors [83]. Thus, LacCer, G_M3_, G_D3_, and G_T3_ are the precursors for the 0-, a-, b-, and c-series gangliosides, respectively [84], and the biosynthesis of these compounds determines the relative proportion of gangliosides in each series (Figure 3).

After the synthesis of these precursors, GalNAc, Gal, and Neu5Ac residues can be transferred in a stepwise manner by the β1,4-*N*-acetylgalactosaminyltransferase I (GM2/GD2 synthase) [86], the β1,3-galactosyltransferase IV [87], and sialyltransferases (Figure 3). The β1,4-*N*-acetyl­galactosaminyltransferase I (β4GalNAc T1) is active on the four series of gangliosides and converts LacCer, G_M3_, G_D3_, and G_T3_ into G_A2_ (asialo-G_M2_), G_M2_, G_D2_, and G_T2_, respectively [88,89]. Similarly, the β1,3-galactosyltransferase IV (β3Gal T4) equally uses G_A2_, G_M2_, G_D2_, and G_T2_ as acceptor substrates [88]. The terminal Gal residue can be further used as acceptor substrate by the α2,3-sialyltransferase ST3Gal II [90]. Finally, the terminal trisaccharide Neu5Acα2-3Galβ1-3­GalNAc can be further substituted by another sialic acid residue in α2,8-linkage by ST8Sia V [83] or in α2,6-linkage to the GalNAc residue by ST6GalNAc V to form α-series gangliosides (Figure 3) [91].

Normal human tissues mainly express “simple” gangliosides from the 0- and a-series, whereas “complex” gangliosides from the b- and c-series are essentially restricted to the nervous system of healthy adults but can be re-expressed in pathological conditions such as melanoma and brain tumors [85]. The regulation of GTs involved in the synthesis of gangliosides is mainly achieved at the transcriptional level and GT gene expression is also tissue-specific [80,92].

### 3.2. Regulation of Ganglioside-Specific Glycosyltransferases by Pro-Inflammatory Cytokines

Chronic inflammation, defined as an aberrant prolonged or dysregulated protective response that occurs in response to the loss of tissue homeostasis, can cause or reinforce several diseases, including diabetes, cancers and neurodegenerative diseases [93]. For instance, the mechanism of tumorigenesis has been associated with an important inflammation at the tumor site environment, mediated by pro-inflammatory cytokines [94,95]. Long-acting harmful environment factors but also intrinsic causes are responsible for the development of these chronic diseases. However, mechanisms by which diseases such as cancers and degenerative diseases develop from chronic inflammation remain largely unknown.

#### 3.2.1. Regulation of Ganglioside Expression During Inflammatory Reactions may be Involved in the Development of Cancers

It is well known that gangliosides such as G_D3_ and G_D2_ are oncofetal markers in neuro-ectoderm derived cancer including melanoma, neuroblastoma and breast cancer, where they play a key role in tumor progression [72,96]. In this context, the modulation of ganglioside expression by pro-inflammatory cytokines is of growing interest. Several studies have reported the impact of inflammatory context on gangliosides expression. First, mice lacking the major receptor for TNF, TNFR1, demonstrated a decreased expression of G_M3_ and G_M1b_ in tissues such as lung, muscle, thymus and spleen [97]. Furthermore, serological analysis of cell surface ganglioside expression in melanocytes incubated with TNF show a higher production of G_M3_ and G_D3_ [98]. In cancer cells, TNF has also been described as a regulator of glycosphingolipid expression. Indeed, it was previously shown that expression of globotriaosylceramide G_b3_, which is considered as a tumor-marker of Burkitt’s lymphomas, is induced on endothelial cells by TNF and IL-1 [99]. Moreover, a study showed that TNF increases G_M2_ ganglioside expression by enhancing the mRNA levels of GM2/GD2 synthase (β4GalNAc T1) in renal carcinoma, contributing to tumor-induced T-cell death [100].

**Figure 4:**
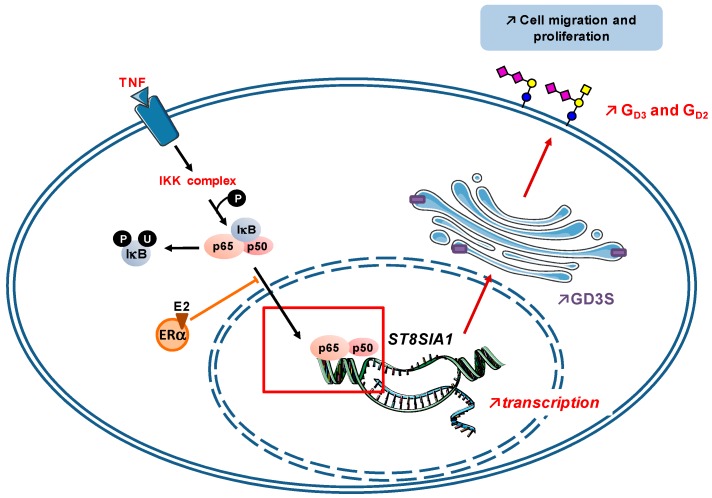
Mechanism of estradiol-dependent repression of GD3S in ER-positive breast cancer cells. GD3S gene (*ST8SIA1*) transcription is activated by NF-κB pathway upon TNF stimulation, leading, in ER-negative breast cancer cells, to an increased expression of GD3S in the Golgi apparatus and disialoganglioside (G_D3_ and G_D2_) at the cell surface, increasing cell migration and proliferation. In ER-positive breast cancer cells, estradiol (E2)-ERα complex represses the TNF-induced up-regulation of GD3S by inhibiting NF-κB nuclear translocation [105].

The disialoganglioside G_D3_, a well-known marker of melanoma and neuroblastoma [72], is also expressed on T-cell leukemia cells while it has been reported to be induced in activated T-cells with various cytokines [101]. More recently, Miyata et al. found that UVB irradiation of keratinocytes, the major source of cytokines in the skin, is able to increase expression of GD3S and GM2/GD2 synthase in melanocytes. Analysis of supernatant composition from keratinocytes revealed that TNF and IL-6 are responsible for enhanced GD3S gene expression [102]. Regulation of ganglioside and their synthetic enzyme might be therefore critical for the promotion of melanoma from activated melanocytes. All together these data indicate that the regulation of ganglioside content by cytokines is closely related to the regulation of the GTs involved in ganglioside biosynthesis. In this prospect, the transcriptional regulation of GD3S gene (*ST8SIA1*), the key enzyme involved in the biosynthesis of b-and c-series gangliosides, has been well described in melanoma, neuroblastoma and breast cancer cells; highlighting a crucial role of NF-κB in activating this gene [103,104,105]. Thus, the TNF-mediated regulation of G_D3_ and G_D2_ previously described could involve the NF-κB pathway, which promotes proliferative and prosurvival gene expression; and has been implicated in EMT, the process by which cancer cells become more invasive and acquire metastatic potential [106]. Interestingly, our studies recently demonstrated that TNF induces up-regulation of the GD3S gene expression in ER-negative breast cancer cells via NF-κB transcription factor while in ER-positive cells, estradiol represses the TNF-induced up-regulation of GD3S by inhibiting NF-κB nuclear translocation (Figure 4) [105]. This result could explain the higher expression of GD3S in ER-negative breast cancer cells and allows a better understanding of the molecular mechanism leading to the regulation of gangliosides expression by cytokines.

#### 3.2.2. Regulatory Function of GSLs in Inflammation and Neurodegeneration

The findings described above suggest that non-infectious inflammatory reactions and especially cytokines expression may be involved in carcinogenesis by regulating the expression of gangliosides that have been considered to be cancer-associated antigens. In the other hand, carbohydrates on the glycosphingolipids expressed on the cell surface membrane play crucial roles in the maintenance of homeostasis by being involved in the fine tuning of cell signaling. It is now well-known that gangliosides are essential for proper nervous system development in vertebrates and deeply involved in the maintenance of the integrity of neural tissues. Indeed, several studies showed that ganglioside defects in KO mice of various glycolipid synthases resulted in the degeneration of nervous system probably due to neuro-inflammation. In this context, Tajima et al. reported in 2010 that DKO mice for GM2/GD2 synthase (β4GalNAc T1) and GD3S led to defective muscarinic acetylcholine receptors expression and aging-related deterioration of cognitive functions [107]. By performing microarray in order to compare gene expression profiles between DKO and WT mice, they later reported that majority of complement component genes and other genes involved in inflammation were up-regulated in the cerebellum but also in the spinal cord of the mutant mice [108]. As it is thought that the complement system and its regulators are implicated in various human neurodegenerative diseases, the regulation of the complement system by gangliosides and subsequent inflammation could be crucial elements for the induction of neurodegeneration in animal models. Moreover, various glycolipid synthase deficiencies have been identified in human families with neurological disorders and especially individuals that exhibited degenerative changes due to inflammation. For instance, a nonsense mutation in GM3 synthase (ST3Gal V) has been reported in individual with infantile-onset symptomatic epilepsy syndrome [109]. More recently, a study identified mutations in the GM2/GD2 synthase enzyme in individuals with hereditary spastic paraplegia [110]. As already mentioned, gangliosides are essential compounds of the plasma membranes where they contribute to the formation of lipid rafts. On the other hand, many of the complement-regulatory proteins, which are GPI-anchored proteins, are localized in lipid rafts [111]. Consequently, alteration in the architectures and functions of these rafts caused by defects of gangliosides might explain how gangliosides defects lead to inflammation and subsequent neurodegeneration.

Gangliosides may also directly interact with immune components and cytokines as demonstrated for ganglioside G_D3_ in the regulation of the pro-inflammatory response of murine microglia mediated by IL-15 [112]. The authors concluded that G_D3_ binds specifically to IL-15, inhibiting the pro-inflammatory effects of the cytokine and thus reducing T-cell proliferation. More recently, a study showed that ganglioside G_D1a_ could inhibit LPS-induced pro-inflammatory cytokines in macrophages by reducing MAPKs and NF-κB signaling pathways through TLR4 [113].

By affecting the immune system, including the regulation of the complement system activation and expression/modulation of cytokines, gangliosides modulate the inflammatory environment and thus may have a direct impact on the control of chronic inflammation, potentially causing the development of neurodegeneration.

### 3.3. Effect of Cell Surface Gangliosides on the Activation of Receptors Tyrosine Kinases and Cell Signaling

The role of gangliosides as regulators of signal transduction was first analyzed by the treatment of cultured cells with exogenous gangliosides directly added in the medium [114]. Numbers of papers have also reported ectopic expression or antisense inhibition strategies targeting specific glycolipid synthases to analyze the role of gangliosides in the regulation of signal transduction. These different approaches clearly demonstrated that gangliosides are fine regulators of receptor tyrosine kinase (RTKs) signaling [115,116]. Several growth factor receptors, including receptors for epidermal growth factor (EGF), fibroblast growth factor (FGF), platelet-derived growth factor (PDGF), hepatocyte growth factor (HGF), nerve growth factor (NGF) or insulin receptors were demonstrated to be positively or negatively regulated by gangliosides [11]. Thus, RTKs are localized in glycolipid-enriched microdomains (GEM) with other lipid rafts associated proteins including integrins and tetraspanins. Changes in gangliosides modify the molecular composition and the structure of glycolipid-enriched microdomains, leading to the reorganization and/or the exclusion of RTKs from GEM [117,118,119]. From a general point of view, it is suggested that monosialogangliosides mostly down-regulated cell signaling whereas disialoglycolipids act as activators of RTKs signaling pathways [120].

#### 3.3.1. Monosialogangliosides Are Negative Sensors of RTKs Signaling

Different studies have demonstrated that monosialogangliosides, especially G_M3_, negatively regulate the activity of RTKs. The negative effect of G_M3_ was first demonstrated for EGFR signaling in a variety of cell lines including hepatoma, hepatocellular carcinoma and neuroblastoma cells [121,122,123,124]. G_M3_ inhibits the transition from inactive EGFR to signaling EGFR dimer, by preventing the autophosphorylation of the intracellular kinase domain in response to ligand binding. It was shown that G_M3_ directly interacts with EGFR on a site distinct from the EGF-binding site through direct carbohydrate-carbohydrate interactions between G_M3_ and EGFR *N*-glycans [125,126,127].

A negative effect of G_M3_ on FGFR activation and tyrosine phosphorylation was also demonstrated in cultured retinal glial cells [128]. G_M3_ depletion by GlcCer synthase inhibition enhances tyrosine phosphorylation of FGFR, activates PI3K/Akt pathway and increases the interactions of FGFR with integrins [129]. In bladder epithelial cells, motility and growth are also modulated by the expression of a-series gangliosides. In the presence of Ca^2+^, G_M3_ and G_M2_ form heterodimers that specifically interact with CD82, impairing the trans-phosphorylation of c-Met receptor, the recruitment of Grb2 and the activation of PI3K/Akt and MEK/Erk pathways [130]. G_M3_ is also implicated in the decrease of vascular endothelial growth factor receptor 2 (VEGFR-2) phosphorylation and subsequent inhibition of Akt downstream signaling pathway in Human Umbilical Vein Endothelial Cells (HUVECs) [131]. G_M3_ decreases VEGF-induced VEGFR-2 activation by blocking receptor dimerization and the binding of VEGF to VEGFR-2 through a G_M3_-specific interaction with the extracellular domain of VEGFR-2 [132].

In parallel to G_M3_, the ganglioside G_M1_ was also shown to negatively regulate RTK signaling. The over-expression of G_M1_ by transfection of β3GalT4 cDNA, the enzyme that converts G_M2_ into G_M1_, inhibited NGF-induced TrkA dimerization and phosphorylation as well as the downstream pathways [133]. In human glioma cells, G_M1_ treatment resulted in reduced PDGFR phosphorylation and signaling, due to the exclusion of the receptor from GEM [134]. The Csk binding protein PAG (Phosphoprotein Associated with Glycosphingolipid-enriched micro-domains) regulates PDGFR partitioning in caveolae and its association with SRC family protein tyrosine kinases (SFK) by controlling G_M1_ levels at the plasma membrane [135].

#### 3.3.2. Disialogangliosides as Activators of RTKs Signaling

Contrasting with the negative effect of monosialogangliosides, disialogangliosides are considered as positive regulators of RTKs signaling. As an example, the introduction of the GD3S gene (*ST8SIA1*) into rat pheochromocytoma PC12 cells resulted in the over-expression of G_D1b_ and G_T1b_. These gangliosides triggered a conformational change of TrkA that formed a constitutively active dimer, activating its downstream signal pathways including Erk1/2 and PI3K/Akt, and leading to a marked enhancement of cell proliferation [136]. Similarly, the expression of the GD3S in MDA-MB-231 breast cancer cells induced the accumulation of b- and c- series gangliosides including G_D3_, G_D2_ and G_T3_ [137,138]. Among these complex gangliosides, G_D2_ was found to be involved in the activation of c-Met, and the subsequent activation of MEK/Erk and PI3K/Akt signaling pathways, leading to enhanced cell migration, proliferation and tumor growth. This was shown by competition assays using anti-G_D2_ mAb that inhibited c-Met phosphorylation, demonstrating the role of the G_D2_ glycan moiety in c-Met activation [138]. Moreover, silencing of the GM2/GD2 synthase efficiently reduced both G_D2_ expression and c-Met phosphorylation. Of importance, the G_D2_-dependent activation of c-Met occurred in the absence of HGF [139]. On the other hand, the disialoganglioside G_D1a_ that belongs to the a-series, was shown to inhibit HGF-induced motility and scattering of mouse osteosarcoma cell variant FBJ-LL cells through the suppression of phosphorylation of c-Met [140]. It was also demonstrated that the interaction of G_D3_ ganglioside with EGF receptor is responsible for sustaining its expression and downstream signaling to maintain the self-renewal of mouse neural stem cells in vitro [141]. Finally, G_D1a_ promotes ligand-independent EGFR dimerization, enhances EGFR-mediated activation of the MAPK signaling pathway [142] and EGFR phosphorylation is significantly reduced with the knockdown of ST3Gal II, the enzyme that converts G_M1_ into G_D1a_ [143].

## 4. Conclusions

At the end of this review, it appears that the glycosylation changes induced by chronic inflammation can be implicated in several diseases. By regulating the expression of specific GTs at the transcriptional level, pro-inflammatory cytokines increase the expression of sialylated glyco-epitopes on mucin *O*-glycans as well as on glycosphingolipids with several consequences.

In the case of chronic inflammation of airways as observed in CF, the increased levels of pro-inflammatory cytokines in response to pathogens lead to a constant NF-κB activation resulting in an increased synthesis of pro-inflammatory cytokines, which contributes to the vicious inflammatory circle. In parallel, activation of NF-κB pathway induces the expression of fucosyl- and sialyltransferases including ST3Gal IV, resulting in the increase of sLe^x^ and 6-sulfo-sLe^x^ expression at the periphery of bronchial mucin *O*-glycan chains. These changes in glycosylation modify the properties of the mucus and create new ligands for pathogens, especially *P. aeruginosa*, increasing again the level of pro-inflammatory cytokines and worsening the disease. Similarly, the mechanism of tumorigenesis is also associated with an important inflammation at the tumor site, mediated by pro-inflammatory cytokines. As demonstrated for melanoma, neuroblastoma and breast cancer, the transcription of the GD3 synthase gene (*ST8SIA1*) is activated by NF-κB pathway upon TNF stimulation. In ER-negative breast cancer cells, it induces the expression of disialogangliosides, especially G_D2_, at the cell surface and a ligand-independent constitutive activation of c-Met, increasing cell migration and proliferation that reinforce the aggressiveness of the pathology.

**Figure 5:**
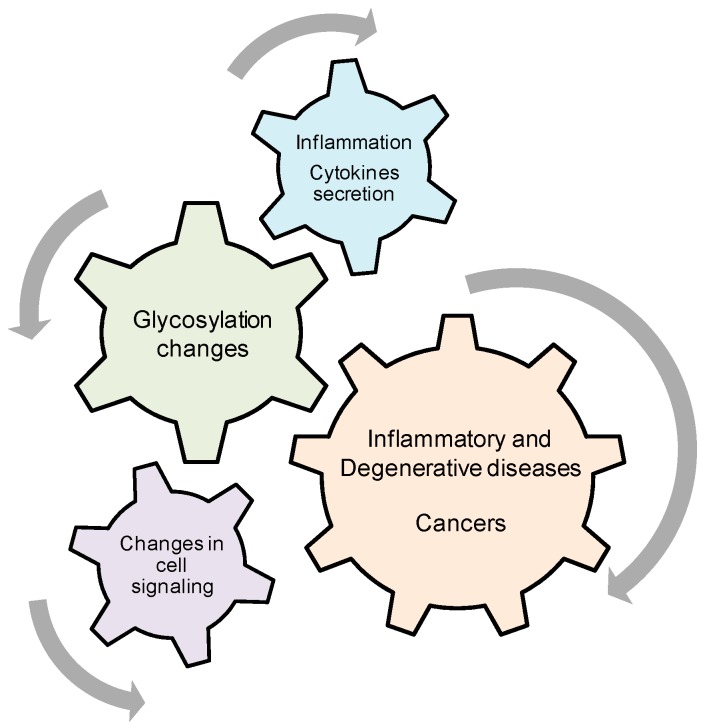
Possible Interplays between Inflammation, Glycosylation, Cell Signaling and Diseases. Pro-inflammatory cytokines induce cell surface glycosylation changes that positively or negatively modulate cell signaling that can cause or reinforce several diseases, including cancers and inflammatory diseases. In the other hand, glycosylation changes can also be involved in the control of inflammation and cytokines secretion.

These two examples clearly illustrate the interplays between inflammation, glycosylation, cell signaling and diseases and underline the need that we have for a better understanding of the mechanisms involved in these relationships (Figure 5). Finally, glycosylation has been recently implicated in the maintenance and the renewal of cancer stem cells. G_D2_ ganglioside has been demonstrated to be an independent marker of breast cancer stem cells [144,145] and GD3 synthase was described to regulate epithelial-mesenchymal transition and metastasis in breast cancer [146]. In that context, it is also reasonable to question about the existence of a relationship between glycosylation, inflammation, epithelial-mesenchymal transition and metastasis.

## Abbreviations

BAL, bronchoalveolar lavages; Cer, ceramide; CF, cystic fibrosis; *CFTR*, cystic fibrosis transmembrane conductance regulator gene; DKO, double knock-out; EGF, epidermal growth factor; ER, estrogen receptor; FGF, fibroblast growth factor; Fuc, fucose; *FUT*, fucosyltransferase gene; Gal, galactose; GalNAc, *N*-acetylgalactosamine; GD3S, GD3 synthase; GEM, glycolipid-enriched microdomains; GI, gastrointestinal; GlcCer, glucosylceramide; GlcNAc, *N*-acetylglucosamine; GT, glycosyltransferase; HGF, hepatocyte growth factor; HUVECs, Human Umbilical Vein Endothelial Cells; IBD, inflammatory bowel diseases; IL, interleukin; IR, insulin receptor; KO, knock-out; LacCer, lactosylceramide; LPS, lipopolysaccharide; MAPK, mitogen-activated protein kinase; Neu5Ac, *N*-acetylneuraminic acid; NF-κB, nuclear factor-κB; NGF, nerve growth factor; nuclear factor-κB; PDGF, platelet-derived growth factor; *P. aeruginosa*, *Pseudomonas aeruginosa*; PDMP, DL-threo-1-Phenyl-2-decanoylamino-3-morpholino-1-propanol; ppGalNAcT, uridine diphosphate-GalNAc: polypeptide-α-*N*-acetylgalactosaminyltransferase; ROS, reactive oxygen species; RTKs, Receptor tyrosine kinases; sLe^x^, sialyl-Lewis^x^; 6-sulfo-sLe^x^, 6-sulfo-sialyl-Lewis^x^; TNF, tumor necrosis factor; TSS, transcription start site; UC, ulcerative colitis; VEGFR-2, vascular endothelial growth factor receptor 2.

## References

[1] Kim Y., Varki A. (1997). Perspectives on the significance of altered glycosylation of glycoproteins in cancer. Glycoconj. J..

[2] Pinho S.S., Reis C.A. (2015). Glycosylation in cancer: Mechanisms and clinical implications. Nat. Rev. Cancer.

[3] Pearce O.M., Läubli H. (2016). Sialic acids in cancer biology and immunity. Glycobiology.

[4] Kawamura Y.I., Adachi Y., Curiel D.T., Kawashima R., Kannagi R., Nishimoto N., Dohi T. (2014). Therapeutic adenoviral gene transfer of a glycosyltransferase for prevention of peritoneal dissemination and metastasis of gastric cancer. Cancer Gene Ther..

[5] Komatsu H., Mizuguchi S., Izumi N., Chung K., Hanada S., Inoue H., Suehiro S., Nishiyama N. (2013). Sialyl Lewis X as a predictor of skip N2 metastasis in clinical stage IA non-small cell lung cancer. World J. Surg. Oncol..

[6] Gakhar G., Navarro V.N., Jurish M., Lee G.Y., Tagawa S.T., Akhtar N.H., Seandel M., Geng Y., Liu H., Bander N.H. (2013). Circulating tumor cells from prostate cancer patients interact with E-selectin under physiologic blood flow. PLoS ONE.

[7] Harduin-Lepers A., Krzewinski-Recchi M.A., Colomb F., Foulquier F., Groux-Degroote S., Delannoy P. (2012). Sialyltransferases functions in cancers. Front. Biosci. (Elite Ed.).

[8] Van Dijk W., Mackiewicz A. (1995). Interleukin-6-type cytokine-induced changes in acute phase protein glycosylation. Ann. N. Y. Acad. Sci..

[9] Delmotte P., Degroote S., Lafitte J.J., Lamblin G., Perini J.M., Roussel P. (2002). Tumor necrosis factor alpha increases the expression of glycosyltransferases and sulfotransferases responsible for the biosynthesis of sialylated and/or sulfated Lewis x epitopes in the human bronchial mucosa. J. Biol. Chem..

[10] Groux-Degroote S., Krzewinski-Recchi M.A., Cazet A., Vincent A., Lehoux S., Lafitte J.J., van Seuningen I., Delannoy P. (2008). IL-6 and IL-8 increase the expression of glycosyltransferases and sulfotransferases involved in the biosynthesis of sialylated and/or sulfated Lewis x epitopes in the human bronchial mucosa. Biochem. J..

[11] Julien S., Bobowski M., Steenackers A., Le Bourhis X., Delannoy P. (2013). How Do Gangliosides Regulate RTKs Signaling?. Cells.

[12] Guérardel Y., Groux-Degroote S., Delannoy P., Cipolla L. (2015). Gangliosphyngolipids: Their structure and biological roles. Carbohydrate Chemistry: State-of-the-Art and Challenges for Drug Development.

[13] Riordan J.R., Rommens J.M., Kerem B., Alon N., Rozmahel R., Grzelczak Z., Zielenski J., Lok S., Plavsic N., Chou J.L. (1989). Identification of the cystic fibrosis gene: Cloning and characterization of complementary DNA. Science.

[14] Mall M.A., Galietta L.J. (2015). Targeting ion channels in cystic fibrosis. J. Cyst. Fibros..

[15] Cystic Fibrosis Mutation Database. http://www.genet.sickkids.on.ca/cftr.

[16] Lakshmanan I., Ponnusamy M.P., Macha M.A., Haridas D., Majhi P.D., Kaur S., Jain M., Batra S.K., Ganti A.K. (2015). Mucins in lung cancer: Diagnostic, prognostic, and therapeutic implications. J. Thorac. Oncol..

[17] Hauber H.P., Foley S.C., Hamid Q. (2006). Mucin overproduction in chronic inflammatory lung disease. Can. Respir. J..

[18] Schulz B.L., Sloane A.J., Robinson L.J., Prasad S.S., Lindner R.A., Robinson M., Bye P.T., Nielson D.W., Harry J.L., Packer N.H. (2007). Glycosylation of sputum mucins is altered in cystic fibrosis patients. Glycobiology.

[19] Copin M.C., Buisine M.P., Devisme L., Leroy X., Escande F., Gosselin B., Aubert J.P., Porchet N. (2001). Normal respiratory mucosa, precursor lesions and lung carcinomas: Differential expression of human mucin genes. Front. Biosci..

[20] Evans C.M., Koo J.S. (2009). Airway mucus: The good, the bad, the sticky. Pharmacol. Ther..

[21] Schjoldager K.T., Clausen H. (2012). Site-specific protein O-glycosylation modulates proprotein processing—Deciphering specific functions of the large polypeptide GalNAc-transferase gene family. Biochim. Biophys. Acta.

[22] Lo-Guidice J.M., Wieruszeski J.M., Lemoine J., Verbert A., Roussel P., Lamblin G. (1994). Sialylation and sulfation of the carbohydrate chains in respiratory mucins from a patient with cystic fibrosis. J. Biol. Chem..

[23] Brockhausen I., Schachter H., Stanley P., Varki A., Cummings R.D., Esko J.D., Freeze H.H., Stanley P., Bertozzi C.R., Hart G.W., Etzler M.E. (2009). O-GalNAc Glycans. Essentials of Glycobiology.

[24] Boat T.F., Kleinerman J.I., Fanaroff A.A., Stern R.C. (1977). Human tracheobronchial secretions: Development of mucous glycoprotein and lysozyme-secreting systems. Pediatr. Res..

[25] Wesley A., Forstner J., Qureshi R., Mantle M., Forstner G. (1983). Human intestinal mucin in cystic fibrosis. Pediatr. Res..

[26] Carnoy C., Ramphal R., Scharfman A., Lo-Guidice J.M., Houdret N., Klein A., Galabert C., Lamblin G., Roussel P. (1993). Altered carbohydrate composition of salivary mucins from patients with cystic fibrosis and the adhesion of *Pseudomonas aeruginosa*. Am. J. Respir. Cell Mol. Biol..

[27] Lamblin G., Boersma A., Lhermitte M., Roussel P., Mutsaers J.H., van Halbeek H., Vliegenthart J.F. (1984). Further characterization, by a combined high-performance liquid chromatography/^1^H-NMR approach, of the heterogeneity displayed by the neutral carbohydrate chains of human bronchial mucins. Eur. J. Biochem..

[28] Xia B., Royall J.A., Damera G., Sachdev G.P., Cummings R.D. (2005). Altered *O*-glycosylation and sulfation of airway mucins associated with cystic fibrosis. Glycobiology.

[29] Davril M., Degroote S., Humbert P., Galabert C., Dumur V., Lafitte J.J., Lamblin G., Roussel P. (1999). The sialylation of bronchial mucins secreted by patients suffering from cystic fibrosis or from chronic bronchitis is related to the severity of airway infection. Glycobiology.

[30] Scharfman A., Degroote S., Beau J., Lamblin G., Roussel P., Mazurier J. (1999). *Pseudomonas aeruginosa* binds to neoglycoconjugates bearing mucin carbohydrate determinants and predominantly to sialyl-Lewis x conjugates. Glycobiology.

[31] Scharfman A., Arora S.K., Delmotte P., van Brussel E., Mazurier J., Ramphal R., Roussel P. (2001). Recognition of Lewis x derivatives present on mucins by flagellar components of *Pseudomonas aeruginosa*. Infect. Immun..

[32] Rhim A.D., Stoykova L.I., Trindade A.J., Glick M.C., Scanlin T.F. (2004). Altered terminal glycosylation and the pathophysiology of CF lung disease. J. Cyst. Fibros..

[33] Khan T.Z., Wagener J.S., Bost T., Martinez J., Accurso F.J., Riches D.W. (1995). Early pulmonary inflammation in infants with cystic fibrosis. Am. J. Respir. Crit. Care Med..

[34] Bonfield T.L., Panuska J.R., Konstan M.W., Hilliard K.A., Hilliard J.B., Ghnaim H., Berger M. (1995). Inflammatory cytokines in cystic fibrosis lungs. Am. J. Respir. Crit. Care Med..

[35] McAllister F., Henry A., Kreindler J.L., Dubin P.J., Ulrich L., Steele C., Finder J.D., Pilewski J.M., Carreno B.M., Goldman S.J. (2005). Role of IL-17A, IL-17F, and the IL-17 receptor in regulating growth-related oncogene-alpha and granulocyte colony-stimulating factor in bronchial epithelium: Implications for airway inflammation in cystic fibrosis. J. Immunol..

[36] Bonfield T.L., Konstan M.W., Berger M. (1999). Altered respiratory epithelial cell cytokine production in cystic fibrosis. J. Allergy Clin. Immunol..

[37] Conese M., Assael B.M. (2001). Bacterial infections and inflammation in the lungs of cystic fibrosis patients. Pediatr. Infect. Dis. J..

[38] Corvol H., Fitting C., Chadelat K., Jacquot J., Tabary O., Boule M., Cavaillon J.M., Clement A. (2003). Distinct cytokine production by lung and blood neutrophils from children with cystic fibrosis. Am. J. Physiol. Lung Cell. Mol. Physiol..

[39] Weber A.J., Soong G., Bryan R., Saba S., Prince A. (2001). Activation of NF-kappaB in airway epithelial cells is dependent on CFTR trafficking and Cl^−^ channel function. Am. J. Physiol. Lung Cell. Mol. Physiol..

[40] Velsor L.W., van Heeckeren A., Day B.J. (2001). Antioxidant imbalance in the lungs of cystic fibrosis transmembrane conductance regulator protein mutant mice. Am. J. Physiol. Lung Cell Mol. Physiol..

[41] Velsor L.W., Kariya C., Kachadourian R., Day B.J. (2006). Mitochondrial oxidative stress in the lungs of cystic fibrosis transmembrane conductance regulator protein mutant mice. Am. J. Respir. Cell Mol. Biol..

[42] Brodlie M., McKean M.C., Johnson G.E., Gray J., Fisher A.J., Corris P.A., Lordan J.L., Ward C. (2010). Ceramide is increased in the lower airway epithelium of people with advanced cystic fibrosis lung disease. Am. J. Respir. Crit. Care Med..

[43] Teichgräber V., Ulrich M., Endlich N., Riethmüller J., Wilker B., de Oliveira-Munding C.C., van Heeckeren A.M., Barr M.L., von Kürthy G., Schmid K.W. (2008). Ceramide accumulation mediates inflammation, cell death and infection susceptibility in cystic fibrosis. Nat. Med..

[44] Verhaeghe C., Remouchamps C., Hennuy B., Vanderplasschen A., Chariot A., Tabruyn S.P., Oury C., Bours V. (2007). Role of IKK and ERK pathways in intrinsic inflammation of cystic fibrosis airways. Biochem. Pharmacol..

[45] Cohen-Cymberknoh M., Kerem E., Ferkol T., Elizur A. (2013). Airway inflammation in cystic fibrosis: Molecular mechanisms and clinical implications. Thorax.

[46] Delmotte P., Degroote S., Merten M., Bernigaud A., van Seuningen I., Figarella C., Roussel P., Perini J.M. (2001). Influence of culture conditions on the α1,2-fucosyltransferase and MUC gene expression of a transformed cell line MM-39 derived from human tracheal gland cells. Biochimie.

[47] Baud V., Karin M. (2001). Signal transduction by tumor necrosis factor and its relatives. Trends Cell. Biol..

[48] Ishibashi Y., Inouye Y., Okano T., Taniguchi A. (2005). Regulation of sialyl-Lewis x epitope expression by TNF-alpha and EGF in an airway carcinoma cell line. Glycoconj. J..

[49] Colomb F., Vidal O., Bobowski M., Krzewinski-Recchi M.A., Harduin-Lepers A., Mensier E., Jaillard S., Lafitte J.J., Delannoy P., Groux-Degroote S. (2014). TNF induces the expression of the sialyltransferase ST3Gal IV in human bronchial mucosa via MSK1/2 protein kinases and increases FliD/sialyl-Lewis(x)-mediated adhesion of *Pseudomonas aeruginosa*. Biochem. J..

[50] Colomb F., Krzewinski-Recchi M.A., El Machhour F., Mensier E., Jaillard S., Steenackers A., Harduin-Lepers A., Lafitte J.J., Delannoy P., Groux-Degroote S. (2012). TNF regulates sialyl-Lewis^x^ and 6-sulfo-sialyl-Lewis^x^ expression in human lung through up-regulation of *ST3GAL4* transcript isoform BX. Biochimie.

[51] Jeffries J.L., Jia J., Choi W., Choe S., Miao J., Xu Y., Powell R., Lin J., Kuang Z., Gaskins H.R. (2016). *Pseudomonas aeruginosa* pyocyanin modulates mucin glycosylation with sialyl-Lewis(x) to increase binding to airway epithelial cells. Mucosal Immunol..

[52] Neurath M.F. (2014). Cytokines in inflammatory bowel disease. Nat. Rev. Immunol..

[53] Enss M.L., Cornberg M., Wagner S., Gebert A., Henrichs M., Eisenblätter R., Beil W., Kownatzki R., Hedrich H.J. (2000). Proinflammatory cytokines trigger MUC gene expression and mucin release in the intestinal cancer cell line LS180. Inflamm. Res..

[54] Elson C.O., Sartor R.B., Tennyson G.S., Riddell R.H. (1995). Experimental models of inflammatory bowel disease. Gastroenterology.

[55] Dkhil M.A., Delic D., Al-Quraishy S. (2013). Goblet cells and mucin related gene expression in mice infected with Eimeria papillata. Sci. World J..

[56] Pullan R.D., Thomas G.A., Rhodes M., Newcombe R.G., Williams G.T., Allen A., Rhodes J. (1994). Thickness of adherent mucus gel on colonic mucosa in humans and its relevance to colitis. Gut.

[57] McCormick D.A., Horton L.W., Mee A.S. (1990). Mucin depletion in inflammatory bowel disease. J. Clin. Pathol..

[58] Raouf A.H., Tsai H.H., Parker N., Hoffman J., Walker R.J., Rhodes J.M. (1992). Sulfation of colonic mucin in ulcerative colitis and Crohn’s disease. Clin. Sci..

[59] Parker N., Tsai H.H., Ryder S.D., Raouf A.H., Rhodes J.M. (1995). Increased rate of sialylation of colonic mucin by cultured ulcerative colitis mucosal explants. Digestion.

[60] Larsson J.M., Karlsson H., Crespo J.G., Johansson M.E., Eklund L., Sjövall H., Hansson G.C. (2011). Altered *O*-glycosylation profile of MUC2 mucin occurs in active ulcerative colitis and is associated with increased inflammation. Inflamm. Bowel Dis..

[61] Munkley J. (2016). The Role of Sialyl-Tn in Cancer. Int. J. Mol. Sci..

[62] Bodger K., Halfvarson J., Dodson A.R., Campbell F., Wilson S., Lee R., Lindberg E., Järnerot G., Tysk C., Rhodes J.M. (2006). Altered colonic glycoprotein expression in unaffected monozygotic twins of inflammatory bowel disease patients. Gut.

[63] Velcich A., Yang W., Heyer J., Fragale A., Nicholas C., Viani S., Kucherlapati R., Lipkin M., Yang K., Augenlicht L. (2002). Colorectal cancer in mice genetically deficient in the mucin Muc2. Science.

[64] Fu J., Wei B., Wen T., Johansson M.E., Liu X., Bradford E., Thomsson K.A., McGee S., Mansour L., Tong M. (2011). Loss of intestinal core 1-derived *O*-glycans causes spontaneous colitis in mice. J. Clin. Investig..

[65] Bergstrom K., Liu X., Zhao Y., Gao N., Wu Q., Song K., Cui Y., Li Y., McDaniel J.M., McGee S. (2016). Defective Intestinal Mucin-Type O-Glycosylation Causes Spontaneous Colitis-Associated Cancer in Mice. Gastroenterology.

[66] Bergstrom K., Fu J., Johansson M.E., Liu X., Gao N., Wu Q., Song J., McDaniel J.M., McGee S., Chen W. (2016). Core 1- and 3-derived *O*-glycans collectively maintain the colonic mucus barrier and protect against spontaneous colitis in mice. Mucosal Immunol..

[67] Hakomori S.I. (2002). The glycosynapse. Proc. Natl. Acad. Sci. USA.

[68] Regina-Todeschini A., Todeschini R.A., Hakomori S.I. (2008). Functional role of glycosphingolipids and gangliosides in control of cell adhesion, motility, and growth, through glycosynaptic microdomains. Biochim. Biophys. Acta.

[69] Birkle S., Zeng G., Gao L., Yu R.K., Aubry J. (2003). Role of tumor-associated gangliosides in cancer progression. Biochimie.

[70] Prokazova N.V., Bergelson L.D. (1994). Gangliosides and atherosclerosis. Lipids.

[71] Ariga T., McDonald M.P., Yu R.K. (2008). Role of ganglioside metabolism in the pathogenesis of Alzheimer’s disease—A review. J. Lipid Res..

[72] Furukawa K., Hamamura K., Aixinjueluo W., Furukawa K. (2006). Biosignals modulated by tumor-associated carbohydrate antigens: Novel targets for cancer therapy. Ann. N. Y. Acad. Sci..

[73] Krengel U., Bousquet P.A. (2014). Molecular recognition of gangliosides and their potential for cancer immunotherapies. Front. Immunol..

[74] Rabu C., McIntosh R., Jurasova Z., Durrant L. (2012). Glycans as targets for therapeutic antitumor antibodies. Future Oncol..

[75] Ichikawa S., Sakiyama H., Suzuki G., Hidari K.I., Hirabayashi Y. (1996). Expression cloning of a cDNA for human ceramide glucosyltransferase that catalyzes the first glycosylation step of glycosphingolipid synthesis. Proc. Natl. Acad. Sci. USA.

[76] Abe A., Inokuchi J., Jimbo M., Shimeno H., Nagamatsu A., Shayman J.A., Shukla G.S., Radin N.S. (1992). Improved inhibitors of glucosylceramide synthase. J. Biochem..

[77] Nomura T., Takizawa M., Aoki J., Arai H., Inoue K., Wakisaka E., Yoshizuka N., Imokawa G., Dohmae N., Takio K. (1998). Purification, cDNA cloning, and expression of UDP-Gal: Glucosylceramide β-1,4-galactosyltransferase from rat brain. J. Biol. Chem..

[78] Takizawa M., Nomura T., Wakisaka E., Yoshizuka N., Aoki J., Arai H., Inoue K., Hattori M., Matsuo N. (1999). cDNA cloning and expression of human lactosylceramide synthase. Biochim. Biophys. Acta.

[79] Zeng G., Yu R.K. (2008). Cloning and transcriptional regulation of genes responsible for synthesis of gangliosides. Curr. Drug Targets.

[80] Ishii A., Ohta M., Watanabe Y., Matsuda K., Ishiyama K., Sakoe K., Nakamura M., Inokuchi J., Sanai Y., Saito M. (1998). Expression cloning and functional characterization of human cDNA for ganglioside GM3 synthase. J. Biol. Chem..

[81] Haraguchi M., Yamashiro S., Yamamoto A., Furukawa K., Takamiya K., Lloyd K.O., Shiku H., Furukawa K. (1994). Isolation of GD3 synthase gene by expression cloning of GM3 α-2,8-sialyltransferase cDNA using anti-GD2 monoclonal antibody. Proc. Natl. Acad. Sci. USA.

[82] Nakayama J., Fukuda M.N., Hirabayashi Y., Kanamori A., Sasaki K., Nishi T., Fukuda M. (1996). Expression cloning of a human GT3 synthase. GD3 and GT3 are synthesized by a single enzyme. J. Biol. Chem..

[83] Kim Y.J., Kim K.S., Do S., Kim C.H., Kim S.K., Lee Y.C. (1997). Molecular cloning and expression of human α-2,8-sialyltransferase (hST8Sia V). Biochem. Biophys. Res. Commun..

[84] Svennerholm L. (1980). Ganglioside designation. Adv. Exp. Med. Biol..

[85] Bobowski M., Cazet A., Steenackers A., Delannoy P. (2012). Role of complex gangliosides in cancer progression. Carbohydr. Chem..

[86] Nagata Y., Yamashiro S., Yodoi J., Lloyd K.O., Shiku H., Furukawa K. (1992). Expression cloning of beta-1,4-*N*-acetylgalactosaminyltransferase cDNAs that determine the expression of GM2 and GD2 gangliosides. J. Biol. Chem..

[87] Amado M., Almeida R., Carneiro F., Levery S.B., Holmes E.H., Nomoto M., Hollingsworth M.A., Hassan H., Schwientek T., Nielsen P.A. (1998). A family of human beta3-galactosyltransferases. Characterization of four members of a UDP-galactose: β-*N*-acetyl­glucosamine/β-*N*-acetyl-galactosamine/β-1,3-galactosyltransferase family. J. Biol. Chem..

[88] Iber H., Zacharias C., Sandhoff K. (1992). The c-series gangliosides GT3, GT2, and GP1c are formed in rat liver Golgi by the same set of glycosyltransferases that catalyze the biosynthesis of asialo-, a- and b-series gangliosides. Glycobiology.

[89] Yamashiro S., Haraguchi M., Furukawa K., Takamiya K., Yamamoto A., Nagata Y., Lloyd K.O., Shiku H., Furukawa K. (1995). Substrate specificity of β-1,4-*N*-acetylgalactosaminyltransferase in vitro and in cDNA-transfected cells. GM2/GD2 synthase efficiently generates asialo-GM2 in certain cells. J. Biol. Chem..

[90] Sturgill E.R., Aoki K., Lopez P.H., Colacurcio D., Vajn K., Lorenzini I., Majic S., Yang W.H., Heffer M., Tiemeyer M. (2012). Biosynthesis of the major brain gangliosides GD1a and GT1b. Glycobiology.

[91] Okajima T., Fukumoto S., Ito H., Kiso M., Hirabayashi Y., Urano T., Furukawa K. (1999). Molecular cloning of brain-specific GD1α synthase (ST6GalNAc V) containing CAG/Glutamine repeats. J. Biol. Chem..

[92] Hidari J.K., Ichikawa S., Furukawa K., Yamasaki M., Hirabayashi Y. (1994). β-1–4-*N*-acetylgalactosaminyl­transferase can synthesize both asialoglycosphingolipid GM2 and glycosphingolipid GM2 in vitro and in vivo: Isolation and characterization of a β-1,4-*N*-acetylgalactosaminyltransferase cDNA clone from rat ascites hepatoma cell line AH7974F. Biochem. J..

[93] Medzhitov R. (2008). Origin and physiological roles of inflammation. Nature.

[94] Coussens L.M., Werb Z. (2002). Inflammation and cancer. Nature.

[95] Elinav E., Nowarski R., Thaiss C.A., Hu B., Jin C., Flavell R.A. (2013). Inflammation-induced cancer: Crosstalk between tumours, immune cells and microorganisms. Nat. Rev. Cancer.

[96] Groux-Degroote S., Guérardel Y., Julien S., Delannoy P. (2015). Gangliosides in breast cancer: New perspectives. Biochemistry (Moscow).

[97] Markotić A., Lümen R., Marusić A., Jonjić S., Müthing J. (1999). Ganglioside expression in tissues of mice lacking the tumor necrosis factor receptor 1. Carbohydr. Res..

[98] Furukawa K., Arita Y., Satomi N., Eisinger M., Lloyd K.O. (1990). Tumor necrosis factor enhances GD3 ganglioside expression in cultured human melanocytes. Arch. Biochem. Biophys..

[99] Van de Kar N.C., Monnens L.A., van Hinsbergh V.W. (1993). Tumor necrosis factor and interleukin 1 induce expression of the glycolipid verotoxin receptor in human endothelial cells. Implications for the pathogenesis of the haemolytic uraemic syndrome. Behring Inst. Mitt..

[100] Raval G., Biswas S., Rayman P., Biswas K., Sa G., Ghosh S., Thornton M., Hilston C., Das T., Bukowski R. (2007). TNFα induction of GM2 expression on renal cell carcinomas promotes T cell dysfunction. J. Immunol..

[101] Yamashiro S., Okada M., Haraguchi M., Furukawa K., Lloyd K.O., Shiku H., Furukawa K. (1995). Expression of alpha 2,8-sialyltransferase (GD3 synthase) gene in human cancer cell lines: High level expression in melanomas and up-regulation in activated T lymphocytes. Glycoconj. J..

[102] Miyata M., Ichihara M., Tajima O., Sobue S., Kambe M., Sugiura K., Furukawa K., Furukawa K. (2014). UVB-irradiated keratinocytes induce melanoma-associated ganglioside GD3 synthase gene in melanocytes via secretion of tumor necrosis factor α and interleukin 6. Biochem. Biophys. Res. Commun..

[103] Kang N.Y., Kim C.H., Kim K.S., Ko J.H., Lee J.H., Jeong Y.K., Lee Y.C. (2007). Expression of the human CMP-NeuAc:GM3 alpha2,8-sialyltransferase (GD3 synthase) gene through the NF-kappaB activation in human melanoma SK-MEL-2 cells. Biochim. Biophys. Acta.

[104] Kwon H.Y., Dae H.M., Song N.R., Kim K.S., Kim C.H., Lee Y.C. (2009). Valproic acid induces transcriptional activation of human GD3 synthase (hST8Sia I) in SK-N-BE(2)-C human neuroblastoma cells. Mol. Cells.

[105] Bobowski M., Vincent A., Steenackers A., Colomb F., van Seuningen I., Julien S., Delannoy P. (2013). Estradiol represses the GD3 synthase gene ST8SIA1 expression in human breast cancer cells by preventing NFκB binding to *ST8SIA1* promoter. PLoS ONE.

[106] Huber M.A., Azoitei N., Baumann B., Grünert S., Sommer A., Pehamberger H., Kraut N., Beug H., Wirth T. (2004). NF-kappaB is essential for epithelial-mesenchymal transition and metastasis in a model of breast cancer progression. J. Clin. Investig..

[107] Tajima O., Egashira N., Ohmi Y., Fukue Y., Mishima K., Iwasaki K., Fujiwara M., Sugiura Y., Furukawa K., Furukawa K. (2010). Dysfunction of muscarinic acetylcholine receptors as a substantial basis for progressive neurological deterioration in GM3-only mice. Behav. Brain Res..

[108] Ohmi Y., Ohkawa Y., Tajima O., Sugiura Y., Furukawa K., Furukawa K. (2014). Ganglioside deficiency causes inflammation and neurodegeneration via the activation of complement system in the spinal cord. J. Neuroinflamm..

[109] Simpson M.A., Cross H., Proukakis C., Priestman D.A., Neville D.C., Reinkensmeier G., Wang H., Wiznitzer M., Gurtz K., Verganelaki A. (2004). Infantile-onset symptomatic epilepsy syndrome caused by a homozygous loss-of-function mutation of GM3 synthase. Nat. Genet..

[110] Boukhris A., Schule R., Loureiro J.L., Lourenço C.M., Mundwiller E., Gonzalez M.A., Charles P., Gauthier J., Rekik I., Acosta Lebrigio R.F. (2013). Alteration of ganglioside biosynthesis responsible for complex hereditary spastic paraplegia. Am. J. Hum. Genet..

[111] Kinoshita T., Fujita M., Maeda Y. (2008). Biosynthesis, remodelling and functions of mammalian GPI-anchored proteins: Recent progress. J. Biochem..

[112] Gómez-Nicola D., Doncel-Pérez E., Nieto-Sampedro M. (2006). Regulation by GD3 of the proinflammatory response of microglia mediated by interleukin-15. J. Neurosci. Res..

[113] Wang Y., Cui Y., Cao F., Qin Y., Li W., Zhang J. (2015). Ganglioside GD1a suppresses LPS-induced pro-inflammatory cytokines in RAW264.7 macrophages by reducing MAPKs and NF-κB signaling pathways through TLR4. Int. Immunopharmacol..

[114] Bremer E.G., Schlessinger J., Hakomori S.I. (1986). Ganglioside-mediated modulation of cell growth. Specific effects of GM3 on tyrosine phosphorylation of the epidermal growth factor receptor. J. Biol. Chem..

[115] Bremer E.G., Hakomori S.I. (1984). Gangliosides as receptor modulators. Adv. Exp. Med. Biol..

[116] Hakomori S., Igarashi Y. (1995). Functional role of glycosphingolipids in cell recognition and signaling. J. Biochem..

[117] Lai A.Z., Abella J.V., Park M. (2009). Crosstalk in Met receptor oncogenesis. Trends Cell Biol..

[118] Park S.Y., Yoon S.J., Freire-de-Lima L., Kim J.H., Hakomori S.I. (2009). Control of cell motility by interaction of gangliosides, tetraspanins, and epidermal growth factor receptor in A431 versus KB epidermoid tumor cells. Carbohydr. Res..

[119] Todeschini A.R., Dos Santos J.N., Handa K., Hakomori S.I. (2007). Ganglioside GM2-tetraspanin CD82 complex inhibits met and its cross-talk with integrins, providing a basis for control of cell motility through glycosynapse. J. Biol. Chem..

[120] Furukawa K., Ohkawa Y., Yamauchi Y., Hamamura K., Ohmi Y., Furukawa K. (2012). Fine tuning of cell signals by glycosylation. J. Biochem..

[121] Huang X., Li Y., Zhang J., Xu Y., Tian Y., Ma K. (2013). Ganglioside GM3 inhibits hepatoma cell motility via down-regulating activity of EGFR and PI3K/AKT signaling pathway. J. Cell. Biochem..

[122] Li Y., Huang X., Zhang J., Li Y., Ma K. (2013). Synergistic inhibition of cell migration by tetraspanin CD82 and gangliosides occurs via the EGFR or cMet-activated Pl3K/Akt signalling pathway. Int. J. Biochem. Cell. Biol..

[123] Coskun Ü., Grzybek M., Drechsel D., Simons K. (2011). Regulation of human EGF receptor by lipids. Proc. Natl. Acad. Sci. USA.

[124] Mirkin B.L., Clark S.H., Zhang C. (2002). Inhibition of human neuroblastoma cell proliferation and EGF receptor phosphorylation by gangliosides GM1, GM3, GD1A and GT1B. Cell Prolif..

[125] Miljan E.A., Meuillet E.J., Mania-Farnell B., George D., Yamamoto H., Simon H.G., Bremer E.G. (2002). Interaction of the extracellular domain of the epidermal growth factor receptor with gangliosides. J. Biol. Chem..

[126] Yoon S.J., Nakayama K., Hikita T., Handa K., Hakomori S.I. (2006). Epidermal growth factor receptor tyrosine kinase is modulated by GM3 interaction with N-linked GlcNAc termini of the receptor. Proc. Natl. Acad. Sci. USA.

[127] Kawashima N., Yoon S.J., Itoh K., Nakayama K. (2009). Tyrosine kinase activity of epidermal growth factor receptor is regulated by GM3 binding through carbohydrate to carbohydrate interactions. J. Biol. Chem..

[128] Meuillet E., Cremel G., Dreyfus H., Hicks D. (1996). Differential modulation of basic fibroblast and epidermal growth factor receptor activation by ganglioside GM3 in cultured retinal Müller glia. Glia.

[129] Toledo M.S., Suzuki E., Handa K., Hakomori S. (2005). Effect of ganglioside and tetraspanins in microdomains on interaction of integrins with fibroblast growth factor receptor. J. Biol. Chem..

[130] Todeschini A.R., Dos Santos J.N., Handa K., Hakomori S.I. (2008). Ganglioside GM2/GM3 complex affixed on silica nanospheres strongly inhibits cell motility through CD82/cMet-mediated pathway. Proc. Natl. Acad. Sci. USA.

[131] Seyfried T.N., Mukherjee P. (2010). Ganglioside GM3 Is Antiangiogenic in Malignant Brain Cancer. J. Oncol..

[132] Chung T.W., Kim S.J., Choi H.J., Kim K.J., Kim M.J., Kim S.H., Lee H.J., Ko J.H., Lee Y.C., Suzuki A. (2009). Ganglioside GM3 inhibits VEGF/VEGFR-2-mediated angiogenesis: Direct interaction of GM3 with VEGFR-2. Glycobiology.

[133] Nishio M., Fukumoto S., Furukawa K., Ichimura A., Miyazaki H., Kusunoki S., Urano T., Furukawa K. (2004). Overexpressed GM1 suppresses nerve growth factor (NGF) signals by modulating the intracellular localization of NGF receptors and membrane fluidity in PC12 cells. J. Biol. Chem..

[134] Mitsuda T., Furukawa K., Fukumoto S., Miyazaki H., Urano T., Furukawa K. (2002). Overexpression of ganglioside GM1 results in the dispersion of platelet-derived growth factor receptor from glycolipid-enriched microdomains and in the suppression of cell growth signals. J. Biol. Chem..

[135] Veracini L., Simon V., Richard V., Schraven B., Horejsi V., Roche S., Benistant C. (2008). The Csk-binding protein PAG regulates PDGF-induced Src mitogenic signaling via GM1. J. Cell Biol..

[136] Fukumoto S., Mutoh T., Hasegawa T., Miyazaki H., Okada M., Goto G., Furukawa K., Urano T. (2000). GD3 synthase gene expression in PC12 cells results in the continuous activation of TrkA and ERK1/2 and enhanced proliferation. J. Biol. Chem..

[137] Cazet A., Groux-Degroote S., Teylaert B., Kwon K.M., Lehoux S., Slomianny C., Kim C.H., Le Bourhis X., Delannoy P. (2009). GD3 synthase overexpression enhances proliferation and migration of MDA-MB-231 breast cancer cells. Biol. Chem..

[138] Cazet A., Bobowski M., Rombouts Y., Lefebvre J., Steenackers A., Popa I., Guérardel Y., Le Bourhis X., Tulasne D., Delannoy P. (2012). The ganglioside GD2 induces the constitutive activation of c-Met in MDA-MB-231 breast cancer cells expressing the GD3 synthase. Glycobiology.

[139] Cazet A., Lefebvre J., Adriaenssens E., Julien S., Bobowski M., Grigoriadis A., Tutt A., Tulasne D., Le Bourhis X., Delannoy P. (2010). GD3 synthase expression enhances proliferation and tumor growth of MDA-MB-231 breast cancer cells through c-Met activation. Mol. Cancer Res..

[140] Hyuga S., Kawasaki N., Hyuga M., Ohta M., Shibayama R., Kawanishi T., Yamagata S., Yamagata T., Hayakawa T. (2001). Ganglioside GD1a inhibits HGF-induced motility and scattering of cancer cells through suppression of tyrosine phosphorylation of c-Met. Int. J. Cancer.

[141] Wang J., Yu R.K. (2013). Interaction of ganglioside GD3 with an EGF receptor sustains the self-renewal ability of mouse neural stem cells in vitro. Proc. Natl. Acad. Sci. USA.

[142] Liu Y., Li R., Ladisch S. (2004). Exogenous ganglioside GD1a enhances epidermal growth factor receptor binding and dimerization. J. Biol. Chem..

[143] Yang H.J., Jung K.Y., Kwak D.H., Lee S.H., Ryu J.S., Kim J.S., Chang K.T., Lee J.W., Choo Y.K. (2011). Inhibition of ganglioside GD1a synthesis suppresses the differentiation of human mesenchymal stem cells into osteoblasts. Dev. Growth Differ..

[144] Battula V.L., Shi Y., Evans K.W., Wang R.Y., Spaeth E.L., Jacamo R.O., Guerra R., Sahin A.A., Marini F.C., Horto-bagyi G. (2012). Ganglioside GD2 identifies breast cancer stem cells and promotes tumorigenesis. J. Clin. Investig..

[145] Liang Y.J., Ding Y., Levery S.B., Lobaton M., Handa K., Hakomori S.I. (2013). Differential expression profiles of glycosphingolipids in human breast cancer stem cells vs. cancer non-stem cells. Proc. Natl. Acad. Sci. USA.

[146] Sarkar T.R., Battula V.L., Werden S.J., Vijay G.V., Ramirez-Peña E.Q., Taube J.H., Chang J.T., Miura N., Porter W., Sphyris N. (2015). GD3 synthase regulates epithelial-mesenchymal transition and metastasis in breast cancer. Oncogene.

